# Transcription activation of circ-STAT3 induced by Gli2 promotes the progression of hepatoblastoma via acting as a sponge for miR-29a/b/c-3p to upregulate STAT3/Gli2

**DOI:** 10.1186/s13046-020-01598-8

**Published:** 2020-06-03

**Authors:** Yanfeng Liu, Jianping Song, Yu Liu, Zhipeng Zhou, Xianqiang Wang

**Affiliations:** 1 Department of Hepatobiliary Surgery, Qilu Hospital of Shandong University, No.107 Wenhuaxi Road, Jinan, 250012 Shandong Province China; 2 Department of General Surgery, 96602 Military Hospital, No.462 Chuanjin Road, Kunming, 650224 Yunnan Province China; 3 Second Department of Hepatobiliary Surgery, PLA General Hospital, No.28 Fuxing Road, Haidian District, Beijing, 100853 China; 4 Department of Pediatric Surgery, PLA General Hospital, No.28 Fuxing Road, Haidian District, Beijing, 100853 China

**Keywords:** Hepatoblastoma, circRNA, STAT3, Gli2, ceRNA

## Abstract

**Background:**

Hepatoblastoma (HB) is a common liver malignancy in children. Our previous study has disclosed the crucial role of STAT3 (signal transducer and activator of transcription 3) in HB.

**Aim of the study:**

Present study was designed to study the circular RNA (circRNA) STAT3 in HB.

**Methods:**

Gel electrophoresis revealed the circular characteristics of circ-STAT3. Function assays like EdU, transwell and sphere formation assay disclosed the function of circ-STAT3 in HB cells. Mechanism assays including ChIP, RIP, RNA pull down assay demonstrated the macular mechanism underlying circ-STAT3.

**Results:**

Circ_0043800, which was originated from STAT3, was up-regulated in HB tissues and cells. More importantly, silencing of circ-STAT3 led to the inhibition on HB cell growth, migration and stem-cell characteristics. Circ_0043800 was predominantly located in the cytoplasm of HB cells. Then, circ_0043800 was found to up-regulate STAT3 via sponging miR-29a/b/c-3p. Besides, we identified that STAT3 overexpression partially rescued silenced circ_0043800, while miR-29a/b/c-3p inhibition completely rescued silenced circ_0043800 on HB cellular biological behaviors. Subsequently, Gli2 (GLI family zinc finger 2) was identified as another target of miR-29a/b/c-3p. Circ_0043800 served as a competing endogenous RNA (ceRNA) to up-regulate both Gli2 and STAT3 via sponging miR-29a/b/c-3p. Moreover, we figured out that Gli2 overexpression completely rescued silenced circ_0043800 on HB cell malignant behaviors. After that, we discovered that Gli2 transcriptionally activated circ_0043800. The in-vivo assays further revealed that circ_0043800 promoted HB tumor growth by up-regulation of Gli2 and STAT3.

**Conclusion:**

Gli2-induced circ_0043800 served as the ceRNA to promote HB by up-regulation of STAT3 and Gli2 at a miR-29a/b/c-3p dependent manner.

## Background

Hepatoblastoma (HB) is a highly invasive malignancy in children and takes up around 50% in pediatric liver cancers [1]. Approximately 20% of HB patients are confronted with metastasis when firstly diagnosed [2]. The annual morbidity of HB is 1.5 cases per million, which represents around 1% in childhood cancers [3], and its incidence has risen by 2.7% each year in the last decades [4]. Patients with lower risk have a 5-year survival rate of 80% while after relapse this number declines to 30–40% [5]. Despite HB control has got advanced due to adjuvant chemotherapy, surgical resection, and liver transplantation, the prognosis for patients with advanced HB remains disappointing [6]. Therefore, it is in need to identify effective biomarkers for early diagnosis of HB.

We have previously published a study that lncRNA LUCAT1 promotes cell proliferation, migration, and invasion in HB via regulation on the miR-301b/STAT3 axis [7]. Thus, present study started from the circRNAs derived from STAT3 (signal transducer and activator of transcription 3) in HB. The main purpose of our current study was to reveal the mechanism of circ-STAT3 in HB progression.

Emerging endogenous circular RNAs (circRNAs) were identified in human cancers [8]. Characterized by the unique loop structure without susceptible 5′ or 3′ ends, circRNAs have strong resistance to exonucleases [9]. Thus, compared with their homologous linear RNA, circRNAs are possessed with greater stability [10]. Present study adopted Sanger sequencing and electrophoresis gel to verify the circular characteristic of circ-STAT3. Due to the stable structure and numerous microRNA (miRNA) binding sites, circRNAs are commonly involved in gene regulation and further influence the occurrence and progression of cancers [11]. As Wang X et al. has revealed, up-regulation of circ_0000517 predicts unfavorable outcomes of patients with hepatocellular carcinoma [12]. CircZKSCAN1 negatively regulates cancer stem cells by competitively binding FMRP to inhibit the binding between FMRP and CCAR1 mRNA and to restrain the Wnt signaling in hepatocellular carcinoma [13]. Circ-0001649 serves as a ceRNA of SHPRH by sponging miR-127-5p, miR-612 and miR-4688, thus inhibiting hepatocellular carcinoma [14]. Circ_0015756 serves as a potential target for HB prognosis, diagnosis, and treatment [15]. CircHMGCS1 binds to the 5’UTR of miR-503-5p to regulate IGF2 and IGF1R expression, further affecting its downstream PI3K-Akt pathway to promote HB cell proliferation and glutaminolysis [16].

It was also reported that circRNAs participate in the competitive endogenous RNA (ceRNA) pattern. The ceRNA pattern means that circRNAs served as the endogenous sponge of miRNAs to block the inhibition of miRNAs on mRNAs. CircRNAs served as the ceRNA to play their tumor facilitator or suppressor roles in multiple cancers. CircPUM1 sponges miR-615-5p and miR-6753-5p to up-regulate NF-κB and MMP2 and to facilitate ovarian cancer tumorigenesis and progression [17]. CircMLLT10 serves as a miR-509-3-5p sponge to antagonize its repressive effect on GINS4, thus accelerating gastric cancer growth and progression [18]. CircFMN2 elevates expression of hTERT via sponging miR-1182 to promote cell proliferation in colorectal cancer [19]. CircRNA GRAMD1B inhibits gastric cancer cell proliferation, migration, and invasion via interaction with miR-130a-3p and regulation on PTEN and p21 expression [20]. In the present study, the interplays between circ-STAT3 and STAT3 as well as other downstream genes were also focused, which might provide a novel regulatory pathway for HB treatment.

## Methods

### Tissue samples collection

Fifty paired hepatoblastoma tissue samples and adjacent non-cancerous tissue samples were collected between January 2014 and March 2019, with the ethical approval from the Ethics Committee of Qilu Hospital of Shandong University and PLA general hospital. Patients did not receive chemotherapy or radiotherapy before surgery. All samples were snap-frozen in liquid nitrogen and stored at -80 °C.

### Cell culture and treatment

Human HB cell lines (HepG2, HuH-6) and human normal liver cell line (THLE-3) used for thus study were available from the Chinese Academy of Sciences (Shanghai, China). DMEM supplying with the 10% FBS and 100 U/ml penicillin/streptomycin was procured from Invitrogen (Carlsbad, CA) for cell culture purposes at 37 °C in 5% CO_2_. RNase R (3 U/μg) was acquired from Epicentre Technologies (Madison, WI).

### Total RNA isolation and quantitative real-time PCR (qRT-PCR)

Total RNAs were extracted from the collected tissues and cultured cells employing Trizol reagent (Invitrogen), PrimeScript™ RT reagent kit (Takara, Shiga, Japan) was then used for generating cDNA. Quantitative analysis was performed with SYBR Premix Ex Taq II (Takara), and the comparative Ct method was applied for data analysis. U6 or GAPDH acted as the normalized gene.

### Fish

Cells of HepG2 and HuH-6 were fixed by 4% formaldehyde and dehydrated, then air-dried for incubation with the circ-STAT3-FISH probe (Ribobio, Guangzhou, China). Following hybridization, cells were rinsed and cultured with Hoechst solution, then analyzed by fluorescence microscope (Olympus Corp., Tokyo, Japan).

### Subcellular fractionation

1 x 10^6^ HB cells were rinsed in PBS, then centrifuged for separating the nuclear RNA and cytoplasmic RNA using the PARIS™ kit (Invitrogen). Expression level of circ-STAT3 was examined by qRT-PCR, with GAPDH and U6 detected as control of cell cytoplasm and cell nuclei, respectively

### Plasmid transfection

The shRNAs and NC-shRNAs were synthesized at Genepharma Company (Shanghai, China) to silence circ-STAT3 and Gli2 in HepG2 and HuH-6 cells utilizing Lipofectamine2000 (Invitrogen). Besides, the miR-29a/b/c-3p mimics/inhibitor and NC mimics/inhibitor, as well as pcDNA3.1/Gli2, pcDNA3.1/STAT3 and NC-pcDNA3.1 were procured from RiboBio. Forty eight h later, transfected cells were reaped for analysis.

### EdU staining

Transfected HB cells were incubated for 3 h with 100 μL of EdU medium diluent in 96-well plate. Cell proliferation was estimated by using EdU staining kit (Ribobio) as per guidebook. Cells were then fixed and stained in DAPI solution, finally observed under Olympus fluorescence microscope.

### Colony formation assay

After transfection, HB cells in 6-well plate were subjected to 14-day cell culture. Then, all cells were first fixed, stained by 0.1% crystal violet, then photographed and analyzed.

### Cell invasion assay

Cell invasion assay was implemented employing the transwell chamber coating with matrigel (BD, Franklin Lakes, NJ). The upper chamber was seeded with 5 × 10^4^ transfected HB cells in serum-free medium, and the lower chamber was supplied with 100% culture medium. Cell invasive ability was examined after 24 h by counting cells on the bottom under microscope.

### Wound-healing assay

5 x 10^6^ HepG2 and HuH-6 cells were plated in the 6-well plate, and then the wounds were created by use of 200-μL sterile tip after reaching about 90% cell density. The cell migration ability was assessed after 24 h under microscope, then photographed.

### Sphere formation assay

The 96-well ultralow attachment plate was commercially acquired from Corning Inc. (New York, NY) for seeding 10 HB cells per well with sphere medium. After 7-day of cell incubation, number of sphere cells was calculated. Sphere efficiency was confirmed by using “the number of cell spheres with diameter greater than 75um in each well” to divide “the total number of original inoculated cells in each well”. Besides, number of cell spheres with diameter greater than 75um in each well was manually calculated under the microscope.

### Western blotting

After lysing in RIPA lysis buffer, protein extracts were separated on 12% SDS-PAGE, shifted to PVDF membranes and cultured with 5% nonfat milk. The primary antibodies (1: 2000; Abcam, Cambridge, MA) against Tubulin, GAPDH and SOX2, Nanog, OCT4, STAT3, Gli2 were used for probing membranes. After washing in TBST, membranes were incubated with the HRP-tagged secondary antibodies (Abcam). ECL detection method (Pierce, Rockford, IL) was applied for detecting protein signals.

### RNA immunoprecipitation (RIP)

Thermo Fisher RIP kit was acquired for performing RIP assay as guided by supplier (Thermo Fisher Scientific, Waltham, MA). In line with the user guide, cell lysates were conjugated with normal control IgG antibody (Millipore) or human Ago2 antibody (Millipore, Billerica, MA) in magnetic beads. The recovered RNAs were examined using qRT-PCR.

### Luciferase reporter assay

The STAT3, circ-STAT3 or Gli2 fragments covering the wild-type and mutated target sites of miR-29a/b/c-3p were synthesized and inserted into the pmirGLO luciferase vector (Promega, Madison, WI). The recombinant vectors were named as STAT3-Wt/Mut, circ-STAT3-Wt/Mut and Gli2-Wt/Mut. The primer sequences for STAT3-Mut were shown as follows: STAT3-Mut (forward): 5-aagtacttagtctaccacgatacaaccttgactccctttctcc-3; STAT3-Mut (reverse): 5-atcgtggtagactaagtacttctcactaaaaggccaatacattac-3. The primer sequences for circ-STAT3-Mut were shown as follows: circ-STAT3-Mut (reverse) 5-gcttaatcgtggtaggaggctgttaactgaag-3; circ-STAT3-Mut (forward) 5-cctcctaccacgattaagcattcagcttccttc-3. T4 ligase was used to link the mutant sequences to the pmirGLO vectors. They were co-transfected into HB cells with miR-29a/b/c-3p mimics or NC mimics for 48 h. For gene promoter analysis, HB cells were co-transfected with the pGL3 luciferase vector (Promega) containing circ-STAT3 promoter, and pcDNA3.1/Gli2 or NC pcDNA3.1. Luciferase activity was examined using Dual-Luciferase Reporter Assay System (Promega).

### Pull down assay

For RNA pull down, cell protein extracts were collected for mixing with the circ-STAT3 biotin probe or circ-STAT3 no-biotin probe as control and magnetic beads using the Pierce Magnetic RNA-Protein Pull-Down Kit as instructed (Thermo Fisher Scientific), following qRT-PCR analysis. For DNA pull down, the protein samples were cultured with Biotin circ-STAT3 promoter or No-biotin circ-STAT3 promoter as control and magnetic beads, following western blot analysis.

### Chromatin immunoprecipitation (ChIP) assay

The fixed HB cells were subjected to 15 min’s crosslink, then treated with ultrasonic for shearing chromatin into 200–1000 base-pair. The Gli2 or control IgG antibody was added into chromatin samples for ChIP assay. After adding beads, the immunoprecipitates were assayed by qRT-PCR.

### Xenograft tumor assay

Xenograft tumor assay was approved by the Animal Health Committee of Qilu Hospital of Shandong University. The male BALB/c nude mice (6 weeks) were commercially and randomly acquired from Beijing Vital River Laboratory (Beijing, China). The transfected HB cells with sh-circ-STAT3#2 or sh-NC were collected for subcutaneous injection into nude mice. For each group, 5 mice were used and a total of 10 mice were used for in-vivo assay. Tumor growth was monitored every 4 days. Nude mice were killed through cervical decapitation after 28-days, tumors were dissected carefully for weigh assessment.

### Immunohistochemistry (IHC)

The tumor tissues collected from the xenograft tumor assay were initially fixed, then dehydrated, embedded in paraffin. Subsequently, the sections (4-μm-thick) were immunostained for IHC assay using specific antibodies to Gli2, STAT3, Nanog, OCT4, PCNA and Ki-67.

### Statistical analyses

Data were all presented as the mean ± standard deviation (SD) from three independent bio-repeats. SPSS (Version 23.0, IBM, Seattle, WA) was carried out for statistical analysis. Difference of groups was analyzed by Student’s t-test or ANOVA, with significant level specified as *p*-value below 0.05.

## Results

### Circ_0043800 was up-regulated in HB tissues and cells

**Fig. 1:**
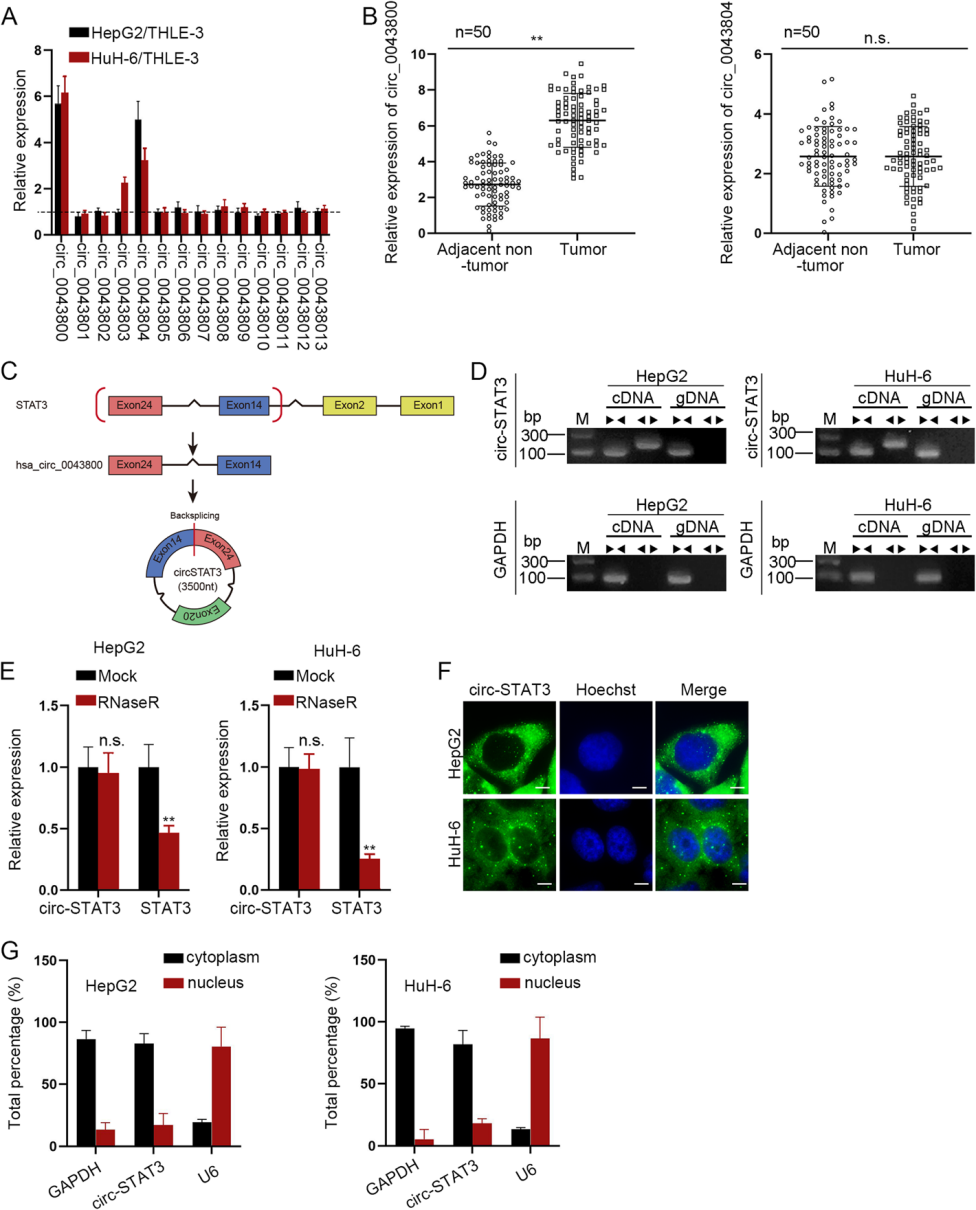
Circ_0043800 was up-regulated in HB tissues and cells. **a** qRT-PCR was applied to measure the expression level of 10 candidate circRNAs in HepG2 and HuH-6 cells (they were referred as HB cells in our study for convenience) compared to control cells of THLE-3. Student’s t-test. **b** qRT-PCR detected relative expression of circ_0043800 and circ_0043804 in HB tissues and adjacent non-tumor tissues. Student’s t-test. **c** Schematic diagram of the splicing pattern of circ_0043800 and Sanger sequencing of circ_0043800. **d** The existence of circ-STAT3 in HB cells was validated by qRT-PCR. **e** Relative expression of circ-STAT3 and STAT3 in HB cells under the treatment of RNaseR. Student’s t-test. **f**-**g** FISH (scale bar: 10 μm) and subcellular fraction assay revealed subcellular location of circ-STAT3. ^**^P < 0.01. The symbol “n.s.” indicates no significance

We firstly detected relative expression of 14 circRNAs which are derived from STAT3 in HepG2 and HuH-6 cells normalized to control cells. The 14 circRNAs were identified by the circBase tool [21]. It was revealed that circ_0043800 and circ_0043804 were highly expressed in HepG2 and HuH-6 cells compared to control cells **(**Fig. 1a**)**. Then, expression of circ_0043800 and circ_0043804 was detected in HB tissues and adjacent non-tumor tissues. The result revealed that only circ_0043800 was up-regulated in HB tissues while circ_0043804 expression showed no significance in tumor tissues and adjacent non-tumor tissues **(**Fig. 1b**)**. Besides, expression of other 12 circRNAs exhibited no significant difference between HB tumor tissues and adjacent non-tumor tissues **(Figure****S1****A)**. We named circ_0043800 as circ-STAT3 for subsequent experiments. As was illustrated in Fig. 1c, circ-STAT3 was formed by the 14th exon and 24th exon. **Figure****S1****B** revealed the Sanger sequencing as well as the backspliced junction of circ-STAT3. Then, convergent primers were used to amplify STAT3 while divergent primers were used for circ-STAT3 with cDNA and gDNA as the templates. It was revealed in qRT-PCR that circ-STAT3 was amplified by divergent primers only in cDNA while STAT3 was amplified by convergent primers in both cDNA and gDNA **(**Fig. 1d**)**. Next, we detected expression of circ-STAT3 and STAT3 by treatment of RNaseR and it was disclosed that RNaseR treatment caused a significant decrease in STAT3 expression while circ-STAT3 expression was not influenced **(**Fig. 1e**)**. Moreover, the subcellular location of circ-STAT3 was identified. It was demonstrated in FISH assay that circ-STAT3 was mainly distributed in the cytoplasm of HB cells **(**Fig. 1f**)**, indicating that circ-STAT3 played a role at post transcription level. Also, subcellular fraction assay revealed that around 80% of circ-STAT3 was distributed in the cytoplasm of HepG2 and HuH-6 cells **(**Fig. 1g**)**. Thus, we determined that circ-STAT3 was upregulated in HB tissues and cells and predominantly located in the cytoplasm.

### Circ-STAT3 promoted HB cell proliferation, invasion, migration and stemness characteristic

**Fig. 2:**
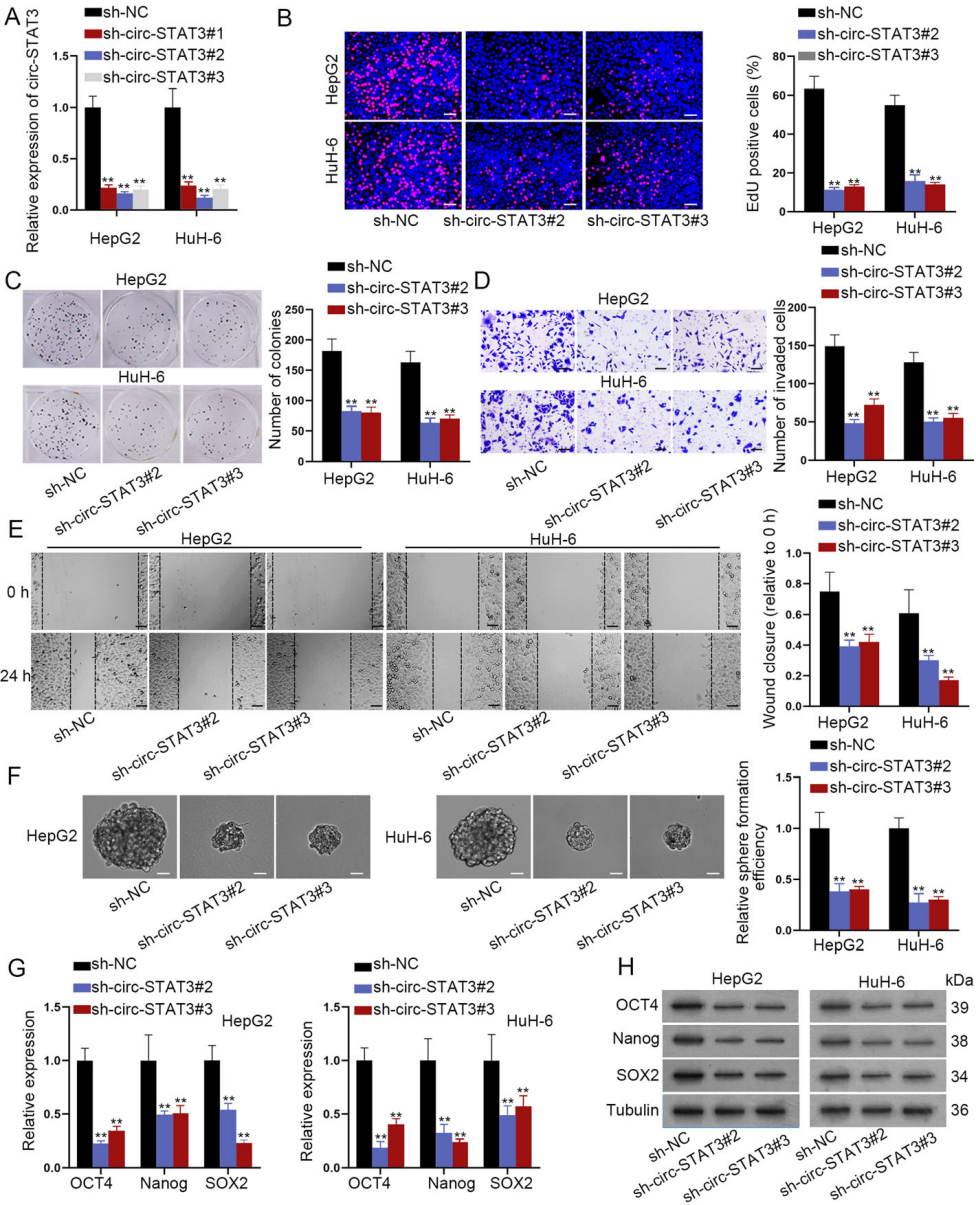
Circ-STAT3 promoted HB cell proliferation, invasion, migration and stemness characteristic. **a** qRT-PCR verified depletion efficiency of circ-STAT3. One-way ANOVA. **b**-**c** EdU (scale bar: 100 μm) and colony formation assay detected proliferation ability of HB cells by knockdown of circ-STAT3. One-way ANOVA. **d**-**e** Transwell (scale bar: 200 μm) and wound healing assay (scale bar: 200 μm) examined invasion and migration ability of cells transfected with sh-circ-STAT3#2/3. One-way ANOVA. **f** Sphere formation assay (scale bar: 200 μm) revealed sphere formation efficiency of circ-STAT3 silenced cells. One-way ANOVA. **g**-**h** qRT-PCR and western blot detected expression of stemness biomarkers in circ-STAT3 silenced cells. One-way ANOVA. ^**^P < 0.01.

Next, function of circ-STAT3 in HB cells was evaluated. The knockdown efficiency of circ-STAT3 was verified for the next loss-of-function assays **(**Fig. 2a**)**. Circ-STAT3 depletion reduced number of EdU positive cells and colonies in EdU staining and colony formation assay **(**Fig. 2b-c**)**. Then, transwell and wound healing assay revealed that number of invaded cells and wound closure were reduced by circ-STAT3 depletion **(**Fig. 2d-e**)**. Further, sphere formation assay revealed lessened sphere formation efficiency in circ-STAT3 silenced cells **(**Fig. 2f**)**. Expression of stemness markers (OCT4, Nanog and SOX2) was decreased by circ-STAT3 silence at the mRNA and protein level **(**Fig. 2g-h**)**. Taken together, knockdown of circ-STAT3 played a suppressive role in HB cell proliferation, invasion, migration and stemness.

### Circ-STAT3 sponged miR-29a/b/c-3p to up-regulate STAT3

**Fig. 3:**
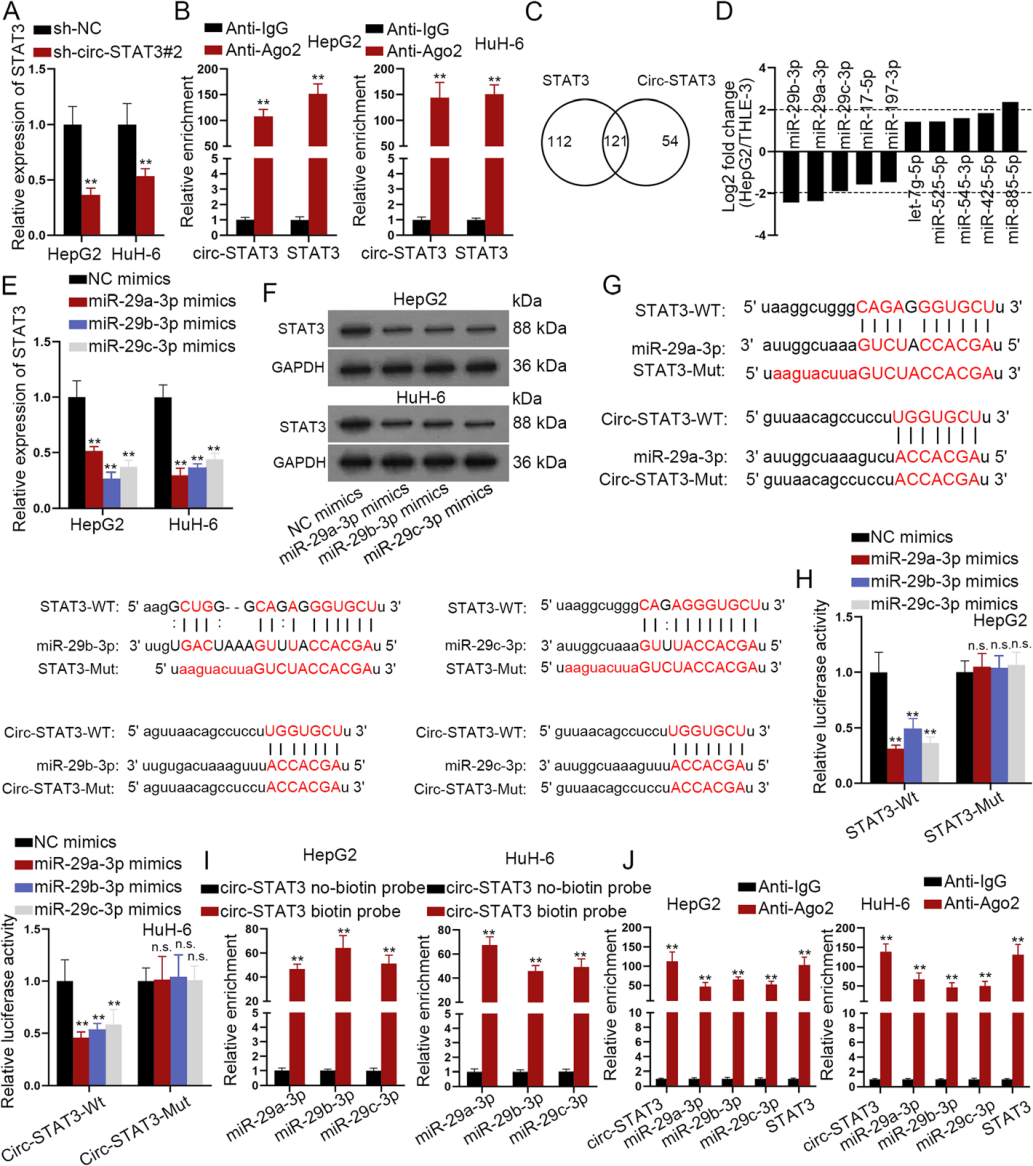
Circ-STAT3 sponged miR-29a/b/c-3p to up-regulate STAT3. **a** Influence of circ-STAT3 depletion on STAT3 expression was evaluated by qRT-PCR analysis. Student’s t-test. **b** RIP assay revealed enrichment of circ-STAT3 and STAT3 pulled down by anti-IgG and anti-Ago2. Student’s t-test. **c** Venn diagram revealed the number of miRNAs binding both circ-STAT3 and STAT3 based on starBase database. **d** The top 10 significantly abnormally expressed miRNAs in HepG2 cells normalized to control cells. **e**-**f** qRT-PCR and western blot examined influence of miR-29a/b/c-3p overexpression on expression of STAT3. One-way ANOVA. **g** Binding sites of wild type/mutant circ-STAT3/STAT3 and miR-29a/b/c-3p. **h** Luciferase reporter assay revealed luciferase activity of wild and mutant circ-STAT3/STAT3 (3 different mutated circ-STAT3/STAT3 with different mutations for miR-29a/b/c-3p) by miR-29a/b/c-3p mimics. One-way ANOVA. **i** RNA pull down assay revealed enrichment of miR-29a/b/c-3p pulled down by biotinylated circ-STAT3 and non-biotinylated circ-STAT3. Student’s t-test. **j** RIP assay revealed enrichment of circ-STAT3, miR-29a/b/c-3p and STAT3 in anti-IgG and anti-Ago2. Student’s t-test. ^**^P < 0.01. The symbol “n.s.” indicates no significance.

Since circular RNAs are widely reported to regulate their host genes, we wondered if circ-STAT3 had the regulatory effects on STAT3 expression. As presented in Fig. 3a, depletion of circ-STAT3 reduced expression of STAT3. Also, circ-STAT3 and STAT3 were significantly enriched in Anti-Ago2 group **(**Fig. 3b**)**, indicating that circ-STAT3 and STAT3 co-existed in RISC. Based on starBase database [22], 121 miRNAs could bind with both circ-STAT3 and STAT3 **(**Fig. 3c**)**. We detected expression of these 121 miRNAs in HepG2 cells and selected the first 10 abnormally expressed miRNAs (5 for up-regulated miRNAs and 5 for down-regulated miRNAs). As was revealed, miR-29a/b/c-3p exhibited the most significant down-regulation in HepG2 cells **(**Fig. 3d**)**. Then, we enhanced expression of miR-29a/b/c-3p in HB cells and the overexpression efficiency of miR-29a/b/c-3p was verified through qRT-PCR analysis **(Figure****S2****A)**. Further, we identified that miR-29a/b/c-3p overexpression significantly reduced expression of STAT3 at the mRNA and protein level **(**Fig. 3e-f**)**. The putative binding sites of STAT3/circ-STAT3 and miR-29a/b/c-3p were predicted from starBase database and were mutated for the following assays **(**Fig. 3g**)**. The luciferase reporter assay disclosed that relative luciferase activity of wild STAT3 and circ-STAT3 was reduced by miR-29a/b/c-3p mimics compared with NC-mimics. In the meanwhile, mutation abrogated the effects of miR-29a/b/c-3p mimics on luciferase activity of STAT3/circ-STAT3 **(**Fig. 3h**)**. Besides, the biotin labeled circ-STAT3 was applied to pull down miR-29a/b/c-3p and it was revealed that miR-29a/b/c-3p was significantly pulled down by biotin labeled circ-STAT3 while no productions were observed in non-biotin labeled circ-STAT3 **(**Fig. 3i**)**. Moreover, the RIP assay revealed that circ-STAT3, miR-29a/b/c-3p and STAT3 were abundantly enriched in Anti-Ago2 precipitated RNA-induced silence complexes (RISCs), while no productions were seen in Anti-IgG **(**Fig. 3j**)**. Besides that, we identified that miR-29a/b/c-3p promoted cell proliferation, invasion, migration and stemness **(Figure****S2****B-E)**. After that, we sought to examine the biological functions of cells expressing circ-STAT3 harboring different mutations for miR-29a/b/c-3p (termed as circ_0043800-Mut1/2/3). **(Figure****S3****A)**. We identified that overexpression of circ_0043800-Mut1/2/3 alone had positive impacts on HB cell proliferation, migration, invasion and stem-like characteristic **(Figure****S3****A-G)**. Whereas, overexpressed circ_0043800 without binding sites with miR-29a/b/c/−3p had no significant effect on above cell functions. More importantly, inhibition of miR-29a/b/c-3p completely reversed the effect of circ_0043800 silencing on cell proliferation (**Figure****S3****H-I**). Based on these data, we concluded that circ-STAT3 served as a ceRNA by sponging miR-29a/b/c-3p.

### STAT3 partially rescued effects in circ-STAT3 on cell biological functions

**Fig. 4:**
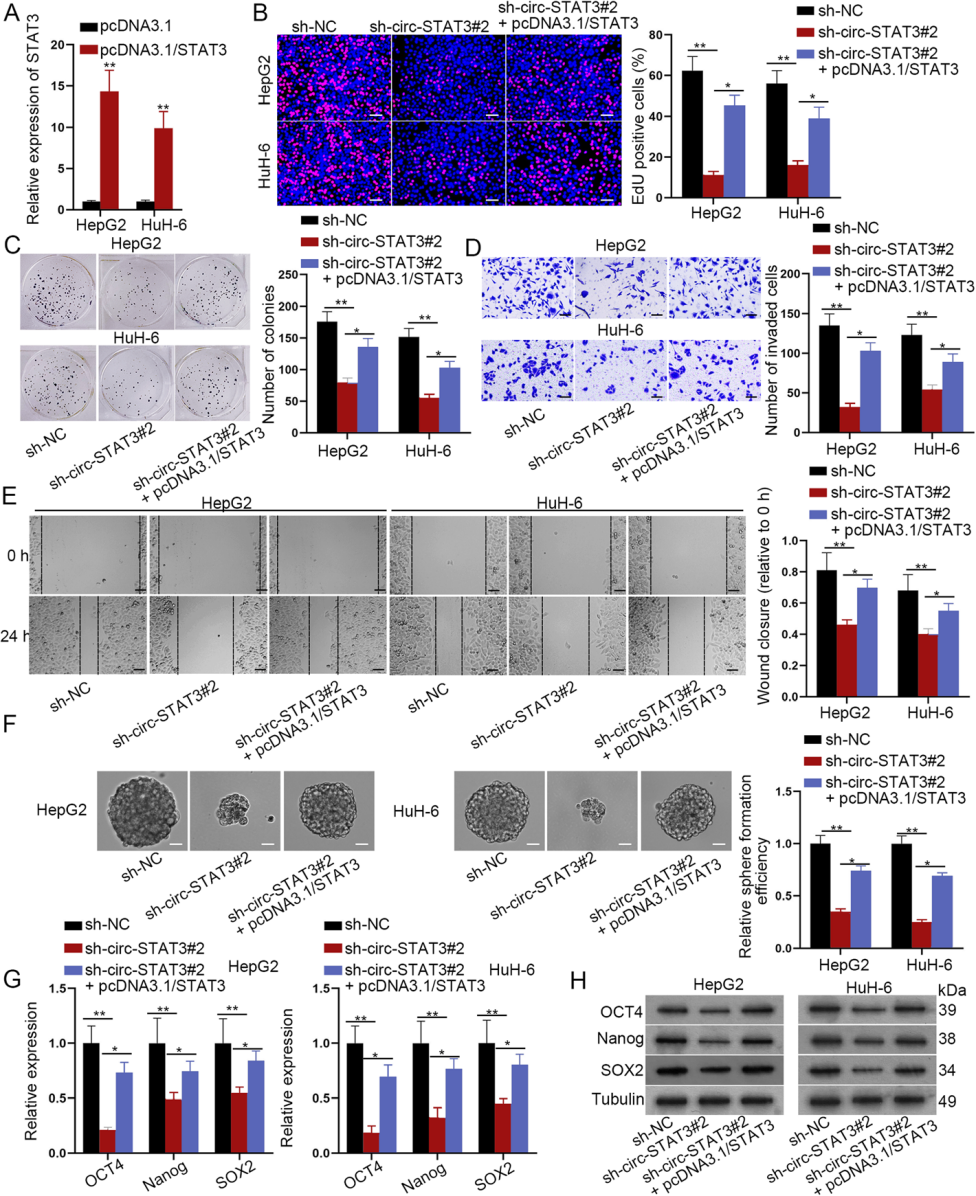
STAT3 partially rescued the effects of circ-STAT3 on cell biological functions. **a** Overexpression efficiency of STAT3 was assessed via qRT-PCR. Student’s t-test. **b**-**c** EdU (scale bar: 100 μm) and colony formation assay detected influence of STAT3 overexpression on circ-STAT3 depletion-mediated proliferation ability of HB cells. One-way ANOVA. **d**-**e** Transwell (scale bar: 200 μm) and wound healing assay (scale bar: 200 μm) examined cell invasion and migration ability in sh-NC, sh-circ-STAT3#2 and sh-circ-STAT3#2+ pcDNA3.1/STAT3 group. One-way ANOVA. **f**-**h** Sphere formation assay (scale bar: 200 μm), qRT-PCR and western blot analysis revealed cell stemness in sh-NC, sh-circ-STAT3#2 and sh-circ-STAT3#2+ pcDNA3.1/STAT3 group. One-way ANOVA. ^*^P < 0.05, ^**^P < 0.01

After we have verified the ceRNA mechanism of circ-STAT3, miR-29a/b/c-3p and STAT3, the functional rescue assays were in need. Overexpression efficiency of STAT3 was firstly verified in qRT-PCR analysis **(**Fig. 4a**)**. The next EdU and colony formation assay revealed that STAT3 up-regulation partially counteracted the suppressive effects of circ-STAT3 depletion on HB cell proliferation ability **(**Fig. 4b-c**)**. Transwell and wound healing assay disclosed that cell invasion and migration ability were reduced by circ-STAT3 silence but was partially rescued by STAT3 up-regulation **(**Fig. 4d-e**)**. Moreover, circ-STAT3 depletion-mediated suppressive influence on HB cell stemness was partially restored by overexpression of STAT3 **(**Fig. 4f-h**)**. In this section, we draw the conclusion that STAT3 partially rescued effects of circ-STAT3 depletion on cell proliferation, invasion, migration and stemness.

### Gli2 was targeted by miR-29a/b/c-3p and was positively regulated by circ-STAT3

**Fig. 5:**
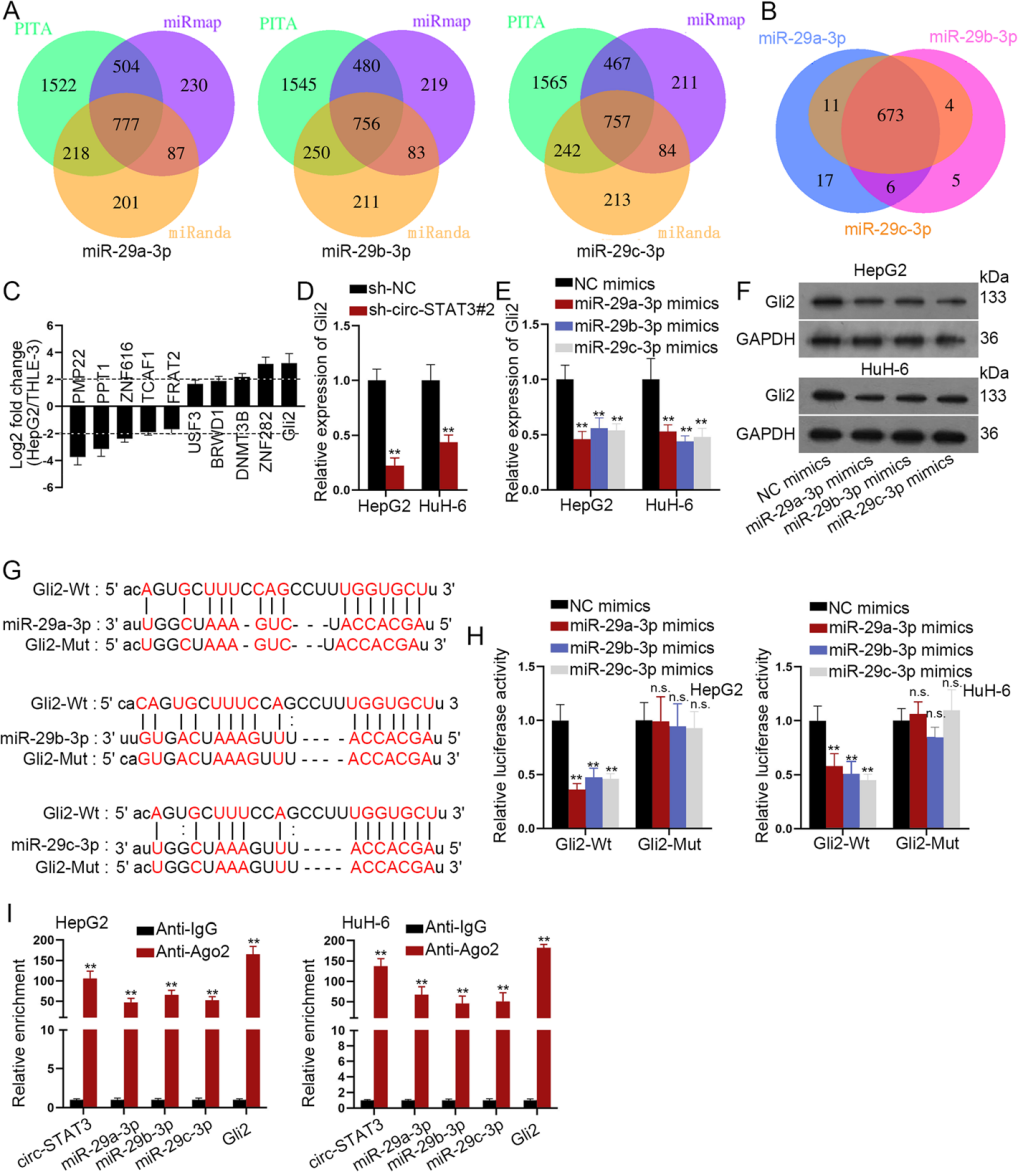
Gli2 was targeted by miR-29a/b/c-3p and was positively regulated by circ-STAT3. **a** Venn diagram revealed the respective number of target genes of miR-29a-3p, miR-29b-3p and miR-29c-3p based on starBase database. **b** The number of common mRNAs which bind with miR-29a-3p, miR-29b-3p and miR-29c-3p. **c** The top 10 significantly abnormally expressed mRNAs in HepG2 cells normalized to control cells. **d** Influence of miR-29a/b/c-3p on expression of Gli2 was evaluated via qRT-PCR. Student’s t-test. **e**-**f** qRT-PCR and western blot examined influence of miR-29a/b/c-3p on expression of Gli2. One-way ANOVA. **g** Binding sites of wild type/mutant Gli2 and miR-29a/b/c-3p. **h** Luciferase activity of wild and mutant Gli2 (three different mutated Gli2 for miR-29a/b/c-3p) by miR-29a/b/c-3p mimics was revealed in luciferase reporter assay. One-way ANOVA. **i** RIP assay of circ-STAT3, miR-29a/b/c-3p and Gli2 in anti-IgG and anti-Ago2. Student’s t-test. ^**^P < 0.01. The symbol “n.s.” indicates no significance

Considering the partial effects of STAT3 on circ-STAT3-mediated cellular function, we explored other targets of miR-29a/b/c-3p. Based on the search results of starBase database. The respective number of target genes for miR-29a-3p (777 targets), miR-29b-3p (756 targets) and miR-29c-3p (757 targets) were illustrated in Fig. 5a. As was shown in Venn diagram, miR-29a/b/c-3p had 673 targets in common **(**Fig. 5b**)**. Expression of these 673 mRNAs in HepG2 cells was evaluated and the top 10 abnormally expressed mRNAs (5 for up-regulated miRNAs and 5 for down-regulated miRNAs) were selected. It was revealed that Gli2 was the most significant up-regulated mRNA with the highest fold change **(**Fig. 5c**)**. Importantly, depletion of circ-STAT3 remarkably reduced expression of Gli2 **(**Fig. 5d**)**. Moreover, up-regulation of miR-29a/b/c-3p caused a noticeable reduce in Gli2 expression at both mRNA and protein levels **(**Fig. 5e-f**)**. Intriguingly, the suppressive effects of miR-29a/b/c-3p on Gli2 were not as significant as that on STAT3. Next, potential binding sequences of miR-29a/b/c-3p and STAT3 were revealed **(**Fig. 5g**)**. We mutated these binding sequences for the follow-up luciferase reporter assay. MiR-29a/b/c-3p overexpression led to the reduced luciferase activity of wild Gli2. When the binding sites were mutated, luciferase activity of Gli2 was not impacted by miR-29a/b/c-3p mimics **(**Fig. 5h**)**. Besides, circ-STAT3, miR-29a/b/c-3p and Gli2 were significantly pulled down by anti-Ago2 but not anti-IgG **(**Fig. 5i**)**. Thus, we concluded that Gli2 was another target of miR-29a/b/c-3p and was positively regulated by circ-STAT3.

### Gli2 completely rescued circ-STAT3 depletion mediated effects on HB cells

**Fig. 6:**
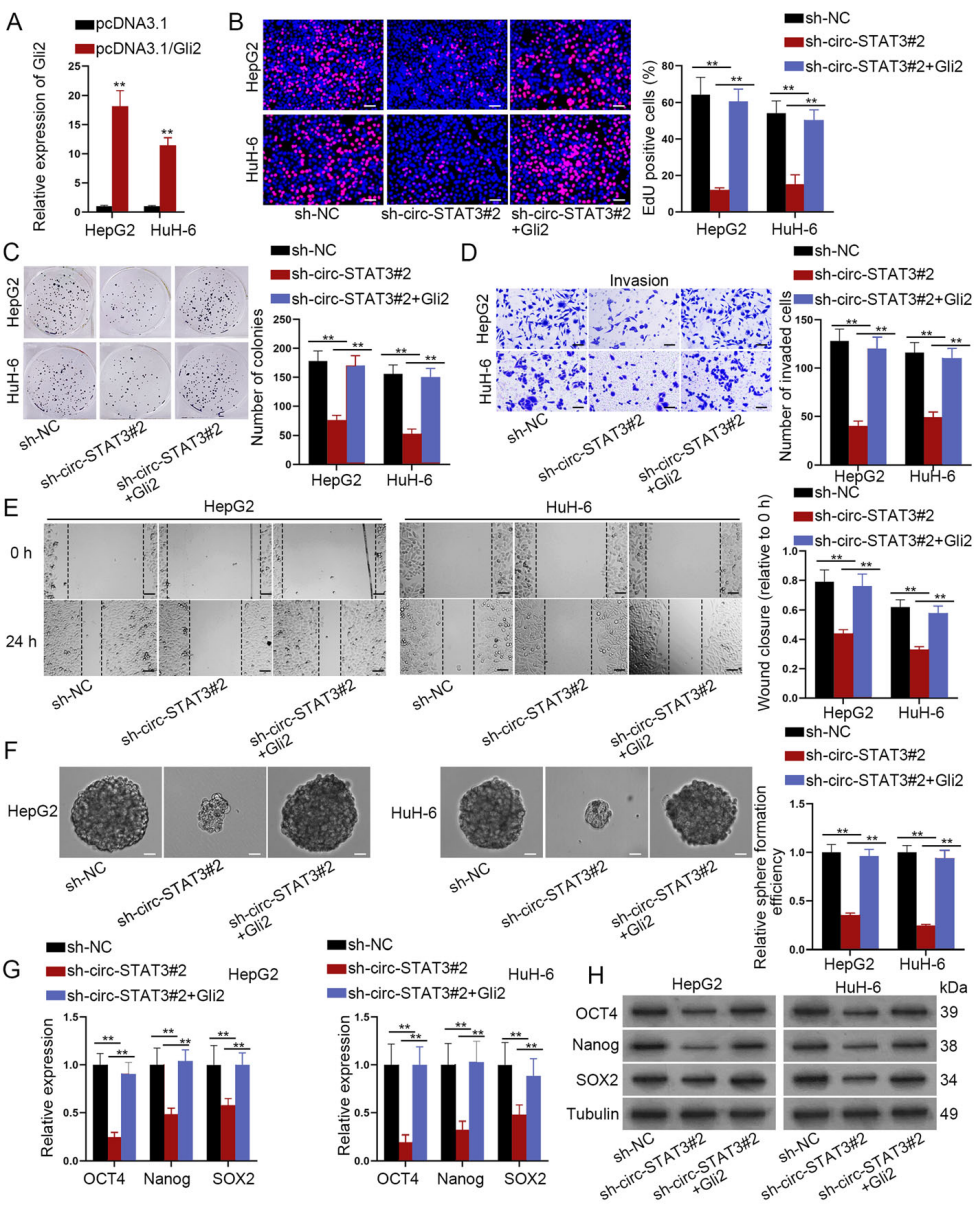
Gli2 completely rescued circ-STAT3 depletion mediated effects on HB cells. **a** qRT-PCR analysis of Gli2 expression in HB cells transfected with pcDNA3.1-Gli2. Student’s t-test. **b**-**c** EdU (scale bar: 100 μm) and colony formation assay detected cell proliferation ability in sh-NC, sh-circ-STAT3#2 and sh-circ-STAT3#2 + pcDNA3.1/Gli2 group. One-way ANOVA. **d**-**e** Transwell (scale bar: 200 μm) and wound healing assay (scale bar: 200 μm) examined influence of Gli2 overexpression on circ-STAT3 depletion-mediated invasion and migration ability of HB cells. One-way ANOVA. **f**-**h** Sphere formation assay (scale bar: 200 μm), qRT-PCR analysis and western blot analysis demonstrated cell stemness characteristic in sh-NC, sh-circ-STAT3#2 and sh-circ-STAT3#2 + pcDNA3.1/Gli2 group. One-way ANOVA. ^**^P < 0.01

After we have identified Gli2 as another target of miR-29a/b/c-3p, whether Gli2 was required in circ-STAT3-mediated HB cell functions need to be further explored. At first, we verified the up-regulation efficiency of Gli2 **(**Fig. 6a**)**. Up-regulation of Gli2 completely rescued the suppressive effects of silenced circ-STAT3 on cell proliferation, invasion and migration ability **(**Fig. 6b-e**)**. Also, silenced circ-STAT3 impaired cell stemness characteristics, but this reduction was completely restored by Gli2 overexpression **(**Fig. 6f-h**)**. In a word, Gli2 took the complete rescue effects in circ-STAT3 on HB cell proliferation, invasion, migration and stemness.

### Gli2 transcriptionally activated STAT3

**Fig. 7:**
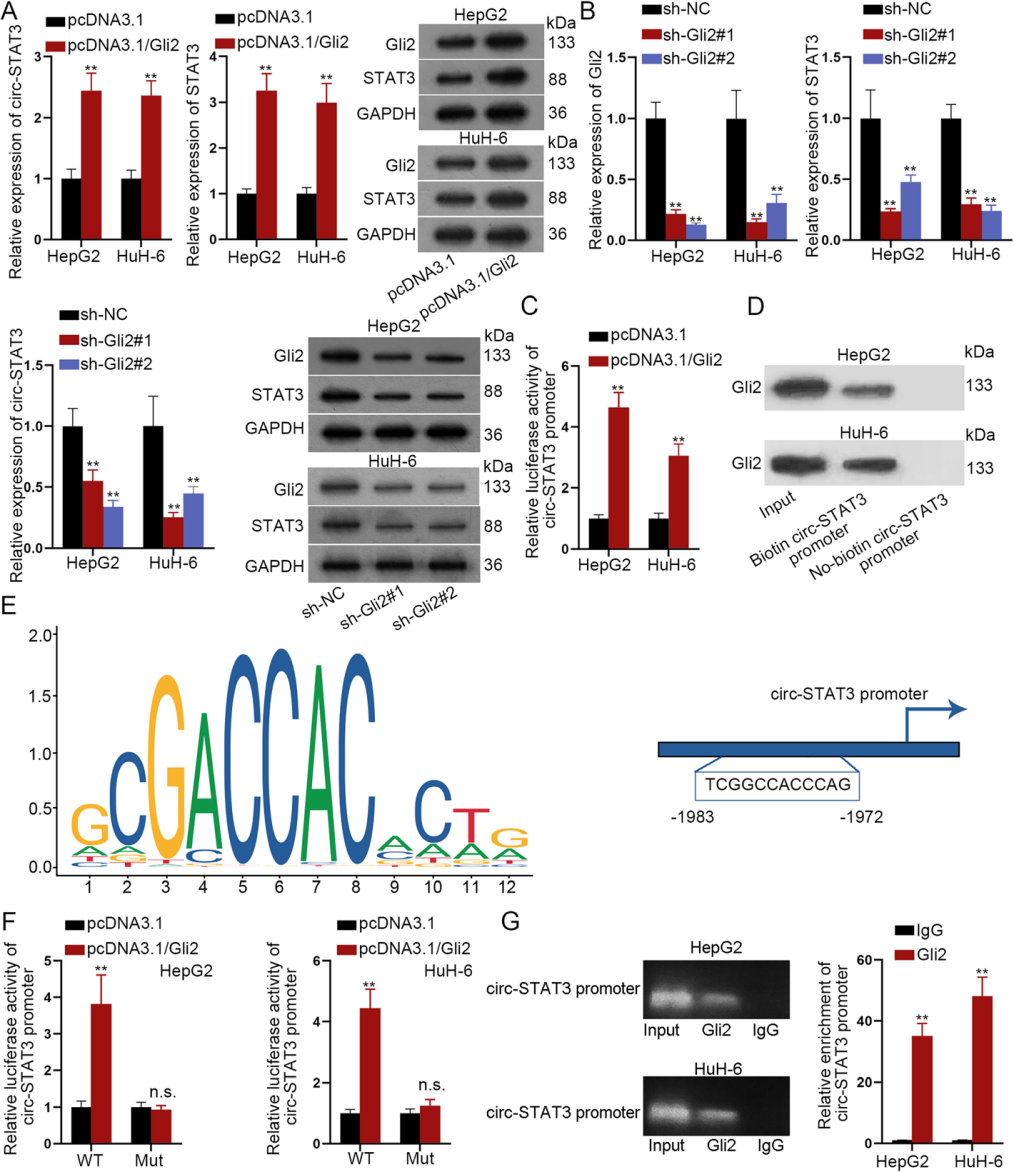
Gli2 transcriptionally activated STAT3. **a** Influence of up-regulated Gli2 on expression of circ-STAT3 and STAT3 were evaluated by qRT-PCR analysis and western blot analysis. Student’s t-test. **b** qRT-PCR and western blot analysis revealed knockdown efficiency of Gli2; influence of down-regulated Gli2 on expression of circ-STAT3 and STAT3 were evaluated by qRT-PCR and western blot analysis. One-way ANOVA. **c**-**d** Luciferase reporter assay and DNA pull-down assay revealed the affinity of Gli2 in circ-STAT3 promoter. Student’s t-test. **e** DNA motif of Gli2 and the binding sites of Gli2 on the promoter of circ-STAT3. **f** Luciferase reporter assay revealed the luciferase activity of wild and mutant circ-STAT3 promoter by up-regulation of Gli2. Student’s t-test. **g** ChIP assay verified relative enrichment of circ-STAT3 promoter pulled down by anti-Gli2. Student’s t-test. ^**^P < 0.01. The symbol “n.s.” indicates no significance

The more significant suppressive effects of miR-29a/b/c-3p on STAT3 than on Gli2 as well as the complete rescue effects of Gli2 on circ-STAT3-mediated cell functions aroused our interest. It has been verified that circRNAs and their host genes could be regulated by the common transcription factor [23], and Gli2 was previously reported as the transcription factor to activate up-regulation of its downstream genes [24]. We wondered whether Gli2 transcriptionally activated STAT3 and further up-regulated circ-STAT3. It was revealed in qRT-PCR and western blot analysis that Gli2 up-regulation elevated the expression of both circ-STAT3 and STAT3 **(**Fig. 7a**)**. Meanwhile, silenced Gli2 significantly reduced the expression of circ-STAT3 and STAT3 **(**Fig. 7b**)**. The following luciferase reporter assay and DNA pull down followed by western blot analysis revealed that Gli2 could bind to the promoter of circ-STAT3 **(**Fig. 7c-d**)**. DNA motif of Gli2 and the putative binding sites of Gli2 on circ-STAT3 promoter were predicted from JASPAR (http://jaspar.genereg.net/) **(**Fig. 7e**)**. We mutated the binding sites and found out that luciferase activity of wild circ-STAT3 promoter was enhanced by the overexpression of Gli2 while mutation led to abolished the increased tendency of luciferase activity **(**Fig. 7f**)**. Accordingly, a ChIP assay revealed that compared with IgG, Gli2 antibody contained the significant enrichment of circ-STAT3 promoter **(**Fig. 7g**)**. According to these data, we concluded that Gli2 was the transcription factor for circ-STAT3 to up-regulate circ-STAT3.

### Circ-STAT3 promoted tumor growth in vivo

**Fig. 8:**
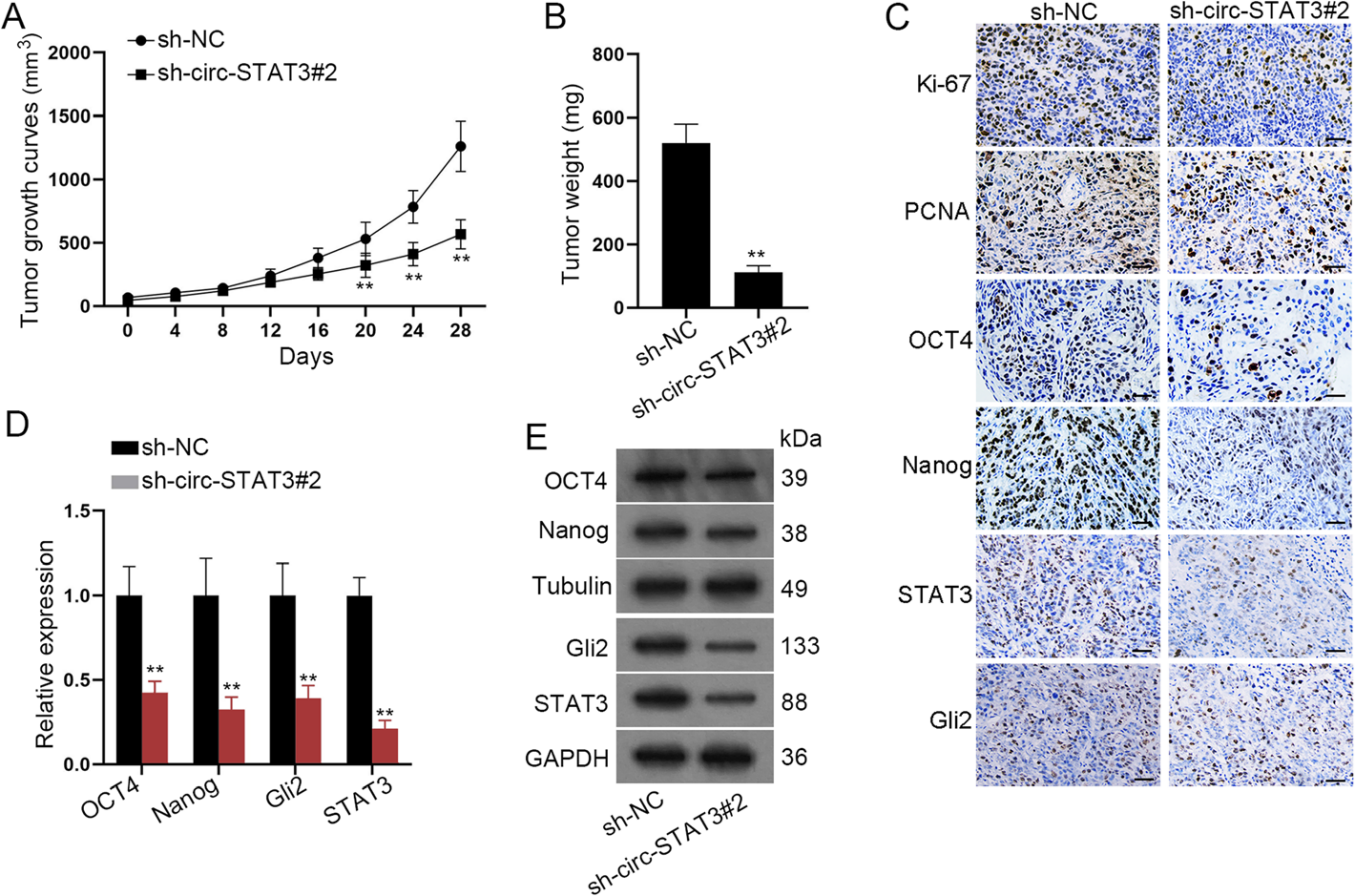
Circ-STAT3 promoted tumor growth in vivo. **a**-**b** Tumor growth curve and tumor weight by depletion of circ-STAT3. Student’s t-test. **c** Immunohistochemistry assay (scale bar: 180 μm) revealed positivity of Ki-67, PCNA, OCT4, Nanog, STAT3 and Gli2. **d**-**e** qRT-PCR and western blot analysis disclosed expression of stemness biomarkers in-vivo. Student’s t-test. ^**^P < 0.01

To further address the effects of circ-STAT3 on HB, the xenograft mouse model was established. As was shown in Fig. 8a-b, circ-STAT3 depletion hindered tumor growth and weight. Also, immunohistochemistry revealed that positivity of Ki-67, PCNA, OCT4, Nanog, STAT3 and Gli2 was decreased by depletion of circ-STAT3 **(**Fig. 8c**)**. Further, we detected mRNA and protein level of stemness biomarkers and the results disclosed that expression of stemness biomarkers was attenuated by silenced circ-STAT3 **(**Fig. 8d-e**)**. In all, circ-STAT3 promoted HB tumor growth in vivo.

Based on all findings, Gli2-induced circ-STAT3 served as a ceRNA against miR-29a/b/c-3p to elevate STAT3 and Gli2 expression, thus facilitating cell proliferation, invasion, migration, stemness and tumor growth in HB **(Figure****S4****)**.

## Discussion

HB is a common malignancy in childhood cancer and has caused a great threat to children health. With the improvement of high throughput sequence analysis, circRNAs have been verified as the crucial contributors in cancer occurrence and progression. However, the role of circRNAs in HB was scarcely reported. STAT3 was a common oncogene in multiple cancers, HB included. Our previous study has disclosed the tumor facilitator role of STAT3 in HB via the ceRNA pattern. Also, STAT3 expression is enhanced by overexpression of NOS3 in Nitric Oxide treated HB cells [25]. Present study was concentrated on the circRNAs which are derived from STAT3 and we found that circ_0043800 was significantly up-regulated in HB cells and tissues. After we have verified the circular features of circ-STAT3, the cytoplasmic role of circ-STAT3 was validated. Next, circ-STAT3 was disclosed to promote HB cell proliferation, invasion, migration and stemness.

CircRNAs were widely reported to regulate their host genes via the ceRNA pattern. For instance, circGFRA1 functions as the ceRNA of GFRA1 by regulating miR-34a in triple negative breast cancer [26]. CircFBLIM1 serves as a ceRNA to regulate FBLIM1 expression via interacting with miR-346 to promote carcinogenesis in hepatocellular cancer [27]. Present study uncovered that circ-STAT3 positively regulated STAT3 and both of them were enriched in the RISCs, indicating that circ-STAT3 might serve as the ceRNA to up-regulate STAT3. The next mechanism assays revealed that circ-STAT3 sponged miR-29a/b/c-3p to up-regulate STAT3. Also, our study disclosed that miR-29a/b/c-3p inhibited cell proliferation, invasion, migration and stemness characteristic. Xiao Z et al. have disclosed that miR-29a-3p inhibits hepatocellular carcinoma cell proliferation and migration via regulation on Mdm2 or PDGF-B [28]. Up-regulation of miR-29a elevates MEG3 expression and hinders cell growth as well as promotes cell apoptosis in hepatocellular cancer [29]. MiR-29b family is proved to inhibit hepatocellular carcinoma cell migration by targeting TET1 [30].

However, it turned out that STAT3 partially rescued circ-STAT3 silence-mediated effects while miR-29a/b/c-3p completely rescued that in HB cells, which indicated that circ-STAT3 might sponge miR-29a/b/c-3p to regulate another mRNA. Subsequently, we identified Gli2 as the down-stream target of miR-29a/b/c-3p. The report of function of Gli2 in HB is limited. However, Gli2 was widely reported in hepatoma. Up-regulation of JCAD inhibits the ability of LATS2 to phosphorylate YAP, thus elevating CCND1 and Gli2 expression to promote hepatoma cell proliferation [31]. Down-regulation of Gli2 plays an anti-cancer role in hepatocellular carcinoma [32]. Down-regulation of Gli2 suppresses cell proliferation and sensitizes hepatocellular carcinoma cells to TRAIL-induced apoptosis [33]. Present study revealed that Gli2 up-regulation completely restored effects of circ-STAT3 on HB cells. Next, we sought to explain this phenomenon. It has been verified that circRNAs and their host genes could be regulated by the common transcription factor [23] and Gli2 was previously verified to transcriptionally activate ARHGEF16 in glioma cells [24]. We wondered if Gli2 served as the transcription factor for circ-STAT3. Such hypothesis was verified in the following assays. Besides that, Gli2 induces transcription of PDGFRB and promotes cancer stem cell properties in gastric cancer [34]. Finally, the in-vivo assay was conducted and the results revealed that circ-STAT3 promoted HB tumor growth via up-regulating STAT3 and Gli2.

The circRNA-involved ceRNA mechanism is scarcely seen in HB but is commonly revealed in liver cancer. As Su Y et al. revealed, circRNA Cdr1as serves as a ceRNA to promote hepatocellular carcinoma progression through sponging miR-1270 to up-regulate AFP [35]. NUDT21 regulates circRNA cyclization and ceRNA crosstalk in hepatocellular carcinoma [36]. CircRNA-104,718 accelerates cell proliferation, migration, invasion, and inhibits apoptosis via miR-218-5p/TXNDC5 axis [37]. Circ_0000567, which is originated from SETD3, inhibits the growth of hepatocellular carcinoma via serving as a ceRNA of MAPK14 through sponging miR-421 [38].

## Conclusion

On the whole, current study revealed that circ-STAT3 served as a sponge of miR-29a/b/c-3p to elevate STAT3 and Gli2 expression with Gli2 as the transcription factor for circ-STAT3. Circ-STAT3 facilitates cell proliferation, invasion, migration, stemness and tumor growth in HB via up-regulation of STAT3 and Gli2, indicating circ-STAT3 as a putative biomarker for HB.

## Supplementary information

**Additional file 1: Figure S1. A.** Relative expression of 12 circRNAs in HB tissues and adjacent non-tumor tissues was assessed via qRT-PCR. Student’s t-test. **B.** Sanger sequencing and backsplice junction of circ-STAT3. The symbol “n.s.” indicates no significance.

**Additional file 2: Figure S2. A.** Overexpression efficiency of miR-29a/b/c-3p was assessed via qRT-PCR. One-way ANOVA. **B-E.** EdU, transwell, wound healing and sphere formation assay revealed the function of miR-29a/b/c-3p upregulation in HB cells. One-way ANOVA. ^**^P < 0.01.

**Additional file 3: Figure S3. A-D.** EdU, colony formation, transwell, wound healing assay exhibited influence of overexpressed circ-STAT3-Mut1, circ-STAT3-Mut2, circ-STAT3-Mut3 or circ-STAT3-Mut1/2/3 on HB cell proliferation, invasion and migration. One-way ANOVA. **E-G.** Sphere formation assay, qRT-PCR and western blot analyses revealed influence of overexpression of circ-STAT3-Mut1/2/3 on HB cell stemness characteristics. One-way ANOVA. **H.** Knockdown efficiency of miR-29a/b/c-3p was verified in qRT-PCR. One-way ANOVA. **I.** EdU assay revealed the rescue effects of miR-29a/b/c-3p inhibitor in circ-STAT3 on cell proliferation. One-way ANOVA. ^**^P < 0.01. The symbol “n.s.” indicates no significance.

**Additional file 4: Figure S4.** Concept map of how circ-STAT3 mediated STAT3 and Gli2 in HB.

## Data Availability

Not applicable.

## Abbreviations

HB: Hepatoblastoma
circRNAs: Circular RNAs
ceRNA: Competing endogenous RNA
miRNA: MicroRNA
STAT3: Signal transducer and activator of transcription 3
Gli2: GLI family zinc finger 2
qRT-PCR: Quantitative real-time PCR
EdU: 5-ethynyl-2′-deoxyuridine
FISH: Fluorescence in situ hybridization
RIP: RNA binding protein immunoprecipitation
ChIP: Chromatin immunoprecipitation
shRNA: Short hairpin RNA
FBS: Fetal bovine serum
DMEM: Dulbecco’s modified eagle’s medium
Rnase R: Ribonuclease R
IHC: Immunohistochemistry
SD: Standard deviation
ANOVA: Analysis of variance
RISC: RNA-induced silencing complex
UTR: Untranslated region
PVDF: Polyvinylidene fluoride
SDS-PAGE: Sodium dodecyl sulfate-polyacrylamide gel electrophoresis
GAPDH: Glyceraldehyde-3-phosphate dehydrogenase
NC: Negative control
