# Supplementary figures and images for: Transcription activation of circ-STAT3 induced by Gli2 promotes the progression of hepatoblastoma via acting as a sponge for miR-29a/b/c-3p to upregulate STAT3/Gli2
Source: J Exp Clin Cancer Res. 2020 Jun 3;39:101. doi: 10.1186/s13046-020-01598-8 (PMC7268652; doi:10.1186/s13046-020-01598-8)

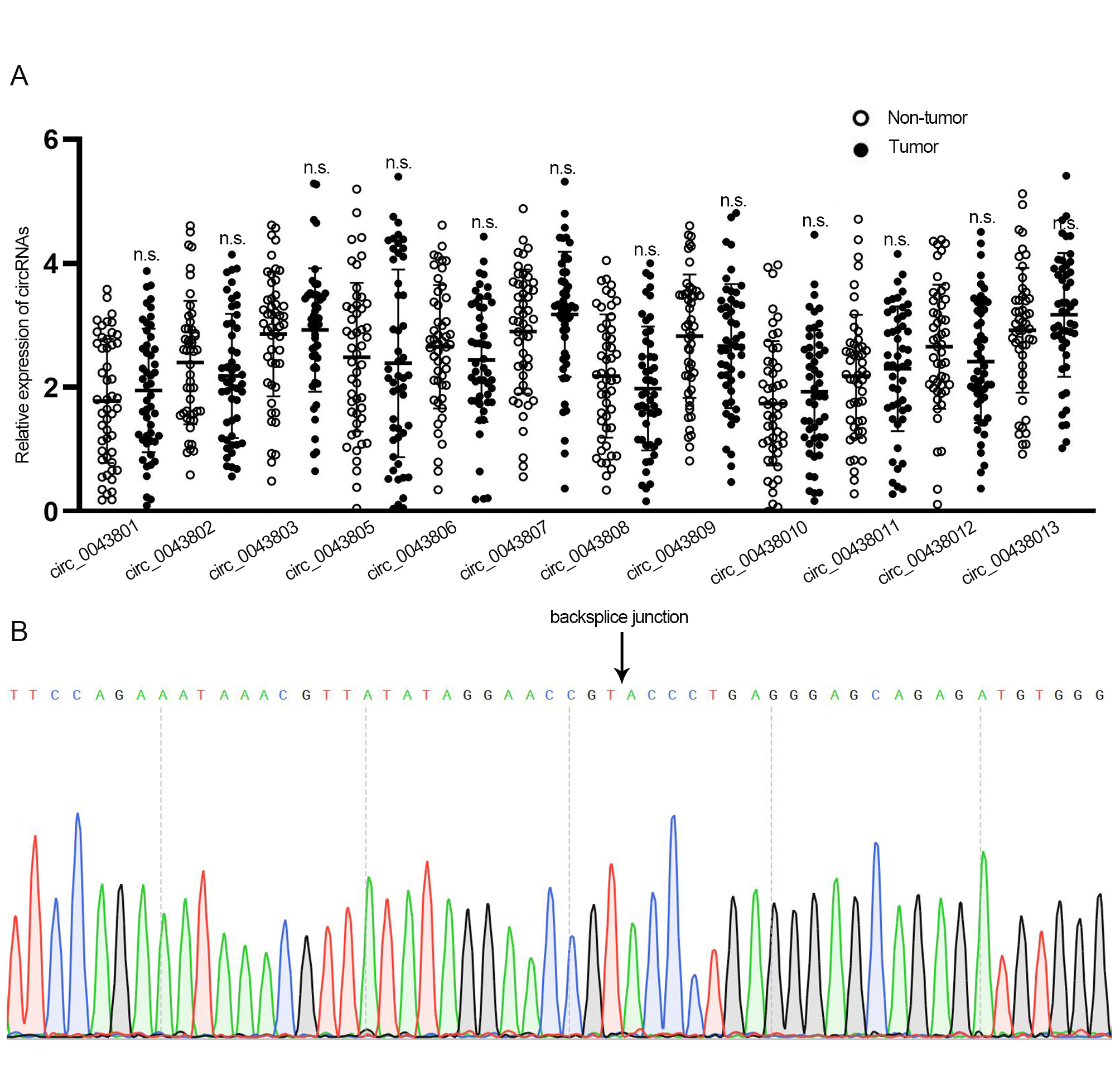

Supplement: Supplementary file 1 — Additional file 1: Figure S1. A. Relative expression of 12 circRNAs in HB tissues and adjacent non-tumor tissues was assessed via qRT-PCR. Student’s t-test. B. Sanger sequencing and backsplice junction of circ-STAT3. The symbol “n.s.” indicates no significance. [file 13046_2020_1598_MOESM1_ESM.tif]

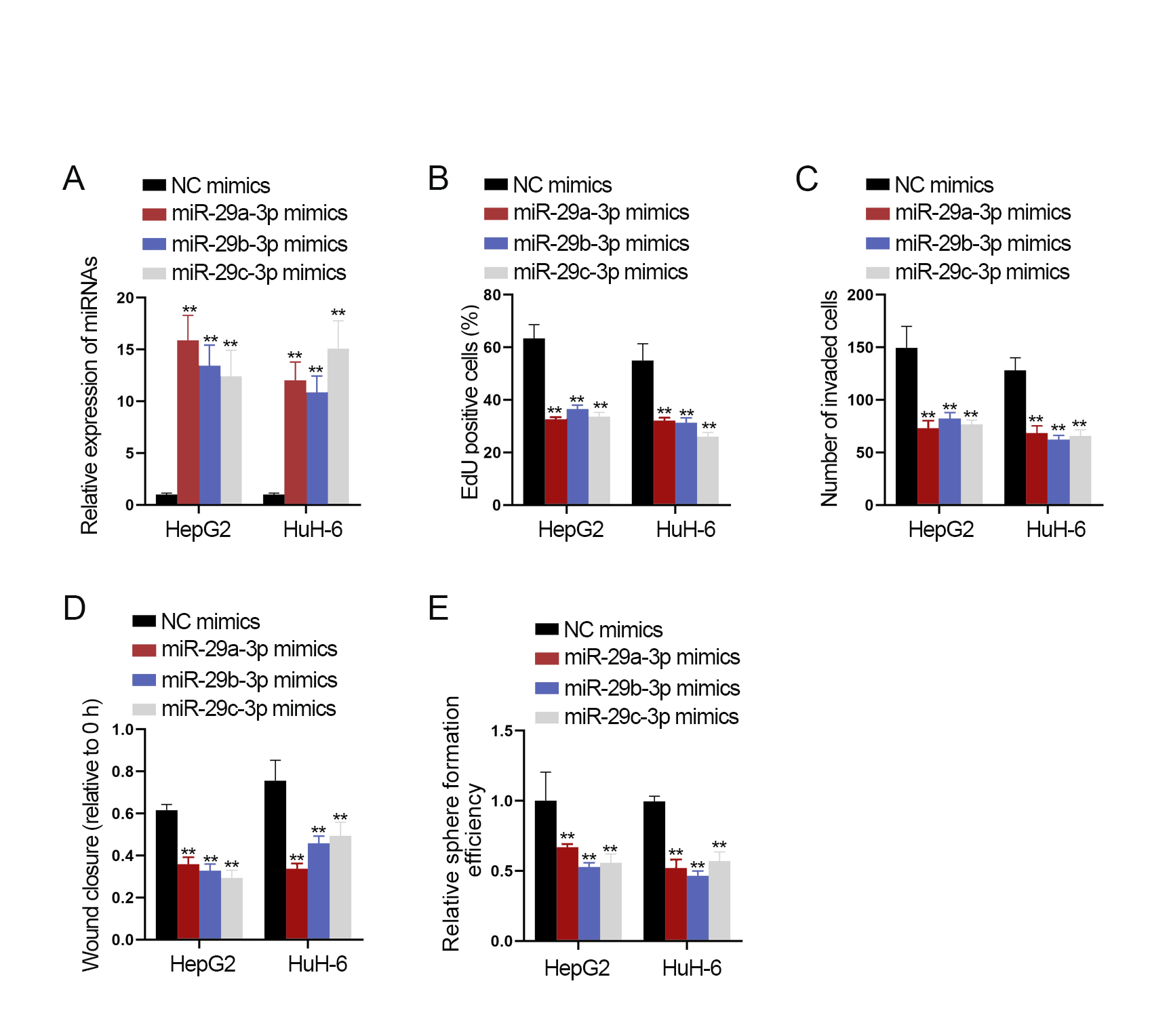

Supplement: Supplementary file 2 — Additional file 2: Figure S2. A. Overexpression efficiency of miR-29a/b/c-3p was assessed via qRT-PCR. One-way ANOVA. B-E. EdU, transwell, wound healing and sphere formation assay revealed the function of miR-29a/b/c-3p upregulation in HB cells. One-way ANOVA. **P < 0.01. [file 13046_2020_1598_MOESM2_ESM.tif]

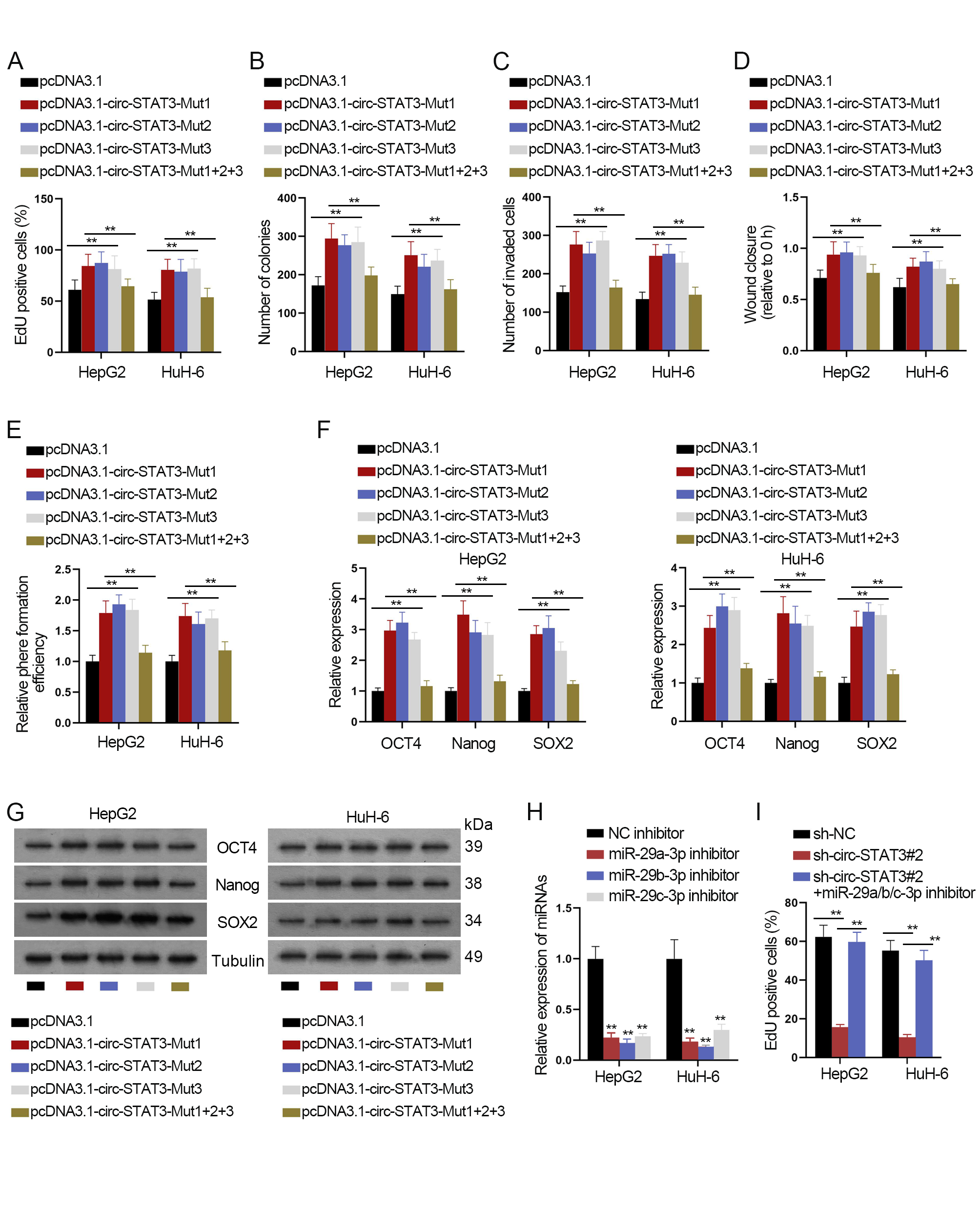

Supplement: Supplementary file 3 — Additional file 3: Figure S3. A-D. EdU, colony formation, transwell, wound healing assay exhibited influence of overexpressed circ-STAT3-Mut1, circ-STAT3-Mut2, circ-STAT3-Mut3 or circ-STAT3-Mut1/2/3 on HB cell proliferation, invasion and migration. One-way ANOVA. E-G. Sphere formation assay, qRT-PCR and western blot analyses revealed influence of overexpression of circ-STAT3-Mut1/2/3 on HB cell stemness characteristics. One-way ANOVA. H. Knockdown efficiency of miR-29a/b/c-3p was verified in qRT-PCR. One-way ANOVA. I. EdU assay revealed the rescue effects of miR-29a/b/c-3p inhibitor in circ-STAT3 on cell proliferation. One-way ANOVA. **P < 0.01. The symbol “n.s.” indicates no significance. [file 13046_2020_1598_MOESM3_ESM.tif]

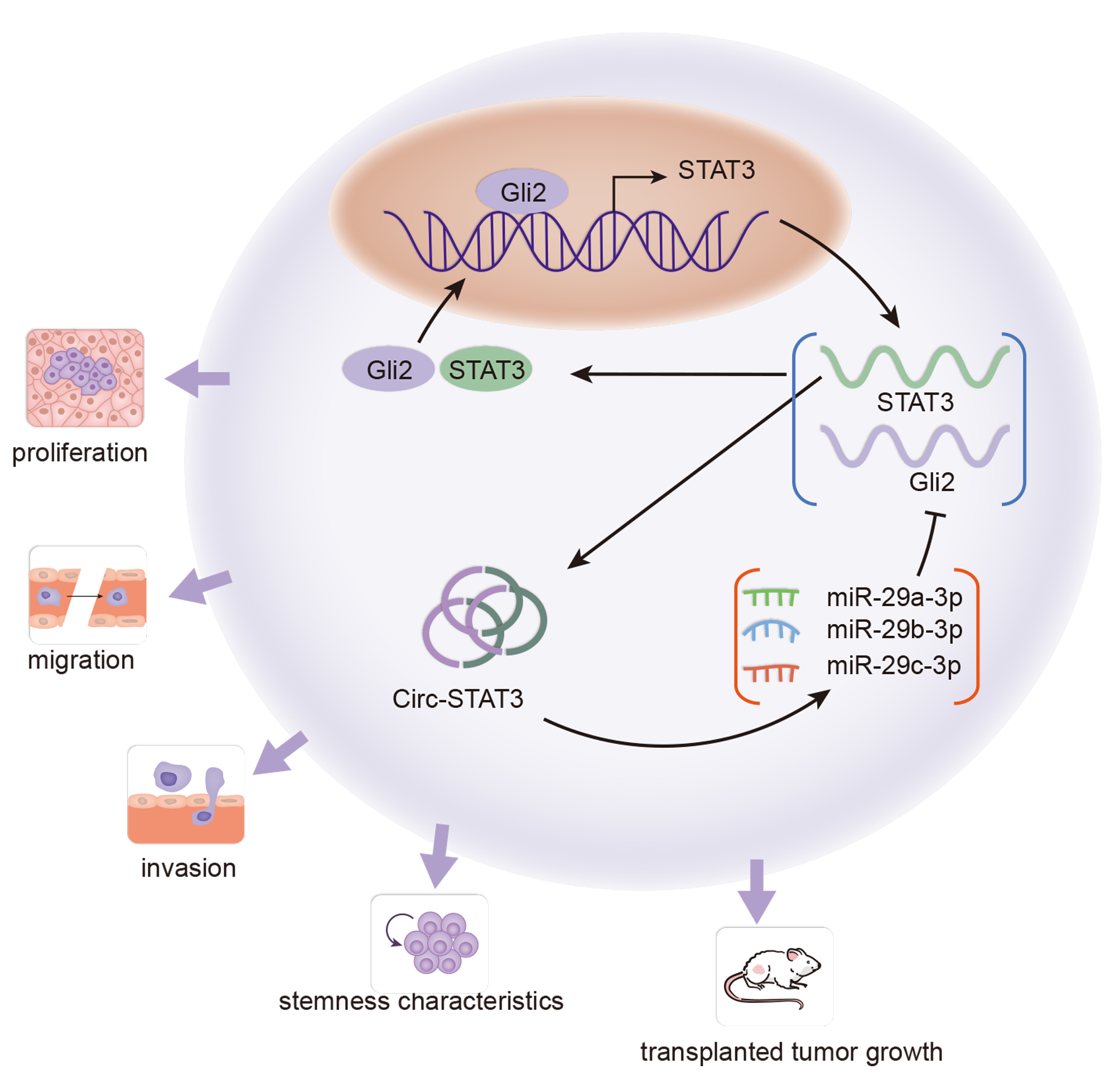

Supplement: Supplementary file 4 — Additional file 4: Figure S4. Concept map of how circ-STAT3 mediated STAT3 and Gli2 in HB. [file 13046_2020_1598_MOESM4_ESM.tif]
